# Quality of Life in SMA Patients Under Treatment With Nusinersen

**DOI:** 10.3389/fneur.2021.626787

**Published:** 2021-03-29

**Authors:** Lucas Mix, Benedikt Winter, Claudia D. Wurster, Sophia Platen, Simon Witzel, Zeljko Uzelac, Heiko Graf, Albert C. Ludolph, Dorothée Lulé

**Affiliations:** ^1^Department of Neurology, Ulm University, Ulm, Germany; ^2^Department of Paediatrics, Ulm University, Ulm, Germany; ^3^Department of Psychiatry and Psychotherapy III, Ulm University, Ulm, Germany; ^4^German Center for Neurodegenerative Diseases Ulm, Ulm, Germany

**Keywords:** patient reported outcome, spinal muscular atrophy, antisense oligonucleotide, nusinersen (Spinraza), quality of life, depressiveness, well-being

## Abstract

**Background:** Spinal Muscular Atrophy (SMA) is a severe neurodegenerative disease, characterized by progressive muscle weakness and atrophy. The approval of the antisense oligonucleotide (ASO) nusinersen now provides an effective pharmacological approach with the potential to slow down or stop disease progression with a potentially major impact on patients' well-being.

**Objective:** This study evaluates quality of life (QoL) in pediatric and adult patients over the course of therapy with nusinersen.

**Methods:** Twenty-six SMA patients treated with nusinersen were evaluated regarding global QoL (gQoL), health-related QoL (HRQoL) and depressiveness. Assessments were conducted three times over the first 6 months of treatment. Applied were different questionnaires: the Anamnestic Comparative Self-Assessment (ACSA) for gQoL, the Short Form-36 Health Survey (SF-36) for HRQoL in adult patients and the ALS Depression Inventory 12 Items (ADI-12) for depressiveness. The sample was matched with 22 healthy controls.

**Results:** Despite severe physical restrictions, patients reported high levels of QoL and low levels of depressiveness at study entry. Early disease onset and low levels of physical functioning were associated with better gQoL and lower levels of depressiveness. A significant decrease of gQoL in patients was evident over the course of the study. Still, adult patients reported a significant increase in perceived health.

**Conclusions:** Our study provides first insight that SMA patients experience a gQoL superior to healthy controls at start of therapy. This might indicate patients' high hopes and expectations toward treatment. gQoL returns to a level similar to that of healthy controls over the course of therapy.

## Introduction

Spinal muscular atrophy (SMA) is a severe inheritable neurodegenerative disease with childhood onset (SMA type I-III) that is typically characterized by progressive muscle weakness and atrophy. For decades, treatment options in SMA were restricted to supportive care. This changed with the approval of the antisense oligonucleotide (ASO) nusinersen for the treatment of 5q-associated SMA types and its considerable efficacy to modify the course of the disease (1). However, current clinical data on the physical outcome mainly rely on investigations in children, whereas evidence on the benefit of nusinersen for older patients is still a subject of current research (2–5).

Despite the tremendous impact of the disease on daily life, previous studies in untreated children and adults with SMA demonstrated a good subjective quality of life (QoL) (6–8). Also, there is no evidence regarding a higher incidence of depressive symptoms in SMA than in the general population (9). One study even reported better mental health in SMA patients with severe motor disability compared to those with milder symptoms (10). In addition, it has been shown, that physical function and self-reported QoL have no association in children and adolescents with SMA (6). A study on children with Duchenne Muscular Dystrophy, a similarly impairing disease with infantile onset, showed that changes in physical function over a period of 12 months did not correlate with changes in Health-related QoL (HRQoL) (11). Satisfactory well-being has not only been reported in SMA, but also in patients with other severe physical restrictions of different etiology (12, 13). This lack of association between physical integrity and mental well-being has been addressed as the “well-being paradox” (12).

Most of the studies investigating QoL in SMA focused on Health-related QoL (HRQoL) (14), which always includes the assessment of physical integrity, instead of focusing on subjective, self-reported measures of QoL (e.g., global QoL). While few studies report on well-being in untreated patients, very little is known about how well-being is affected by therapy with nusinersen. Some studies within the clinical trials of nusinersen reported on quality of life in treated children. Here, a slight but not significant increase in HRQoL was observed after 85 days of treatment with nusinersen in children with SMA types II and III in the age between 2 and 4 years (15). Children with SMA types II and III in the age between 2 and 12 years reported a significantly better HRQoL after 15 months of treatment compared to an untreated sham-control group (16). These findings were confirmed by an open-lable extension study (17).

These studies provide an evidence regarding beneficial effects of nusinersen on QoL in children. Recent studies also investigated the clinical efficacy of the ASO on physical performance in treated adolescents and adults with promising results (3–5). A further study focusing on patient-reported outcomes showed that confidence in the treatment with nusinersen was maintained (over a period of 10 months) (2). However, to our best knowledge, there is no study that elucidates the impact of the treatment with the ASO nusinersen on QoL in adolescents and adults with SMA. We therefore conducted a clinical study on 26 predominantly adolescent and adult patients with SMA under treatment with nusinersen. Global QoL (gQoL), Health-related QoL (HRQoL) and depressive symptoms were evaluated over a period of 6 months.

## Methods

### Participants

We included a total of *N* = 26 SMA (type I: *N* = 4, type II: *N* = 9, type III: *N* = 13) patients with genetically confirmed deletion in exon 7 and/or 8 in the SMN1 gene, who received treatment with nusinersen at the Department of Neurology, Ulm University Hospital (Germany). Of those, *N* = 14 were adult and *N* = 12 pediatric patients with a median age of 44.2 and 13.4 years, respectively. While *N* = 6 classified as “walkers,” *N* = 7 were “sitters” and *N* = 13 “non-sitters.” In addition, we included a control group of 22 neurologically healthy individuals mean-matched for sex, age and education.

For the evaluation of well-being at study entry, data of all participants were analyzed. For the assessment of well-being over 6 months, only those patients with complete data sets for all three time points of investigation were included in the final analyses. One patient presented with pre-diagnosed depression. To avoid potential confounding in the study, this patient was included for the anaylsis of prevalence of depressive symptoms at study entry, but was then excluded from all further analyses. A detailed description of all drop-outs is provided in Table 1.

**Table 1:** Demographics and clinical characteristics.

			**Total**	**Adult**	**Pediatric**
*n* (individuals)			26	14	12
Age (years)	Median		23.4	44.2	13.4
	u. quart.		45.4	48.9	15.3
	l. quart.		13.5	30.0	11.5
	Min.		7.5	21.9	7.5
	Max.		60.8	60.8	17.7
Disease onset (years)	Median		1.0	1.0	0.0
	u. quart.		3.0	13.5	2.0
	l. quart.		0.0	0.0	0.0
	Min.		0.0	0.0	0.0
	Max.		17.0	17.0	3.0
Sex (individuals)	Male		16	10	6
	Female		10	4	6
Type of SMA (individuals)	I		4	0	4
	II		9	5	4
	III		13	9	4
Motor function (individuals)	Walker		6	4	2
	Sitter		7	4	3
	Non-sitter		13	6	7
*n* after drop outs	gQoL	Day 1	24^a^^,^^b^		
		Day 180	21^a^^,^^b^^,^^c^^,^^d^^,^^e^		
	HRQoL	Day 1		12^b^^,^^h^	
		Day 180		11^b^^,^^c^^,^^g^^,^^h^	
	Dep.	Day 1	21^a^^,^^h^		
		Day 180	15^a^^,^^b^^,^^c^^,^^d^^,^^h^^,^^i^^,^^j^		
	Physical function (HFMSE/ ALSFRS-R)	Day 1	22^k^/26		
		Day 180	21^k^^,^^l^/25^l^		

*u. quart., upper quartile; l. quart., lower quartile; min., minimum; max., maximum; HFMSE, Hammersmith Functional Motor Scale Expanded with values from 0 (minimum physical function) to 66 (maximum physical function); ALSFRS-R, ALS Functional Rating Scale Revised with values from 0 (minimum physical function) to 48 (maximum physical function); gQoL, Global quality of life; HRQoL, Health-related quality of life; dep., depressiveness*.

a *One patient included after day 1*.

b *One patient with pre-diagnosed depression was included exclusively for evaluation of the prevalence of depressive symptoms with ADI-12 at day 1*.

c *One adult patient wished to end participation in the study after day 1*.

d *One pediatric patient wished to end participation in the study after day 1*.

e *Data collection missed in one pediatric patient*.

f *Two adult patients were included after day 60*.

g *One pediatric patient turning 18 in the course of the study was included in the assessment with SF-36*.

h *Refrained from data collection at day 1 in 4 pediatric patients in the patient's interest*.

i *Data collection missed in 2 adult patients*.

j *One patient wished to end assessment with ADI-12 after day 1*.

k *The 4 patients with type I SMA were not tested with HFMSE*.

l *One pediatric patient could no longer be tested for physical function due to a change of clinic after day 60*.

The study was approved by the ethics committee of Ulm University (Approval Number 19/12). Written informed consent was obtained from all participants (or their legal guardians).

### Design

The study addressed the assessment of physical function and psychological well-being, including QoL and depressiveness. The interviews took place at day 1, day 60, and day 180 (after 6 months) of treatment.

### Well-Being

QoL was evaluated as global QoL (gQoL) with Anamnestic Comparative Self-Assessment (ACSA) (18) for all patients and as Health-related QoL (HRQoL) with Short Form 36 Health Survey (SF-36) (19) for adult patients. SF-36 was also used for one patient turning 18 in the year of assessment. This study also included the assessment of HRQoL in pediatric patients with PedsQL (20). However, due to unfortunate circumstances during data acquisition, we were only able to collect PedsQL data from 8 out of the 12 pediatric patients. Thus, we excluded this questionnaire from our final analyses, but provide the corresponding data in our Supplementary Materials.

The ACSA is a self-anchored rating scale for subjective overall QoL that is thought to be largely independent of age or other sociodemographic parameters (21). Subjects were asked to rate their overall QoL of the last 2 weeks on a scale ranging from −5 to +5 (−5 = worst ever individually experienced QoL; 0 = neutral state; +5 = best ever personally experienced QoL).

The SF-36 is a self-reporting scale for the assessment of perceived HRQoL within the past 4 weeks and comprises 36 items on a 5- or 6-point Likert scale. Assessed are eight different dimensions of HRQoL: physical functioning, role-limitations due to physical health, bodily pain, (perception of) general health, vitality, social functioning, role-limitations due to emotional status and mental health. In addition, the (perceived) health transition compared to the last year is evaluated as a single item measure.

Depressive symptoms were assessed using the ALS Depression Inventory 12 Items (ADI-12) (22), a questionnaire designed for the use in patients with neurodegenerative diseases. The questionnaire, referring to the past 2 weeks, comprises 12 questions with answers on a 4-point Likert scale and addresses anhedonia, mood and energy (22). ADI-12 does not evaluate any symptoms to which both neurodegenerative disease or depression could have contributed (22). Younger children were spared the question if “they often wished they were dead” of the ADI-12. To make them comparable, scores (normally ranging from 12 to 48) were uniformly transformed to percentages of the maximum score (ranging from 25 to 100%).

All questionnaires were applied in fashion of a semi-structured interview.

### Clinical Data

Physical function was evaluated with a semi-structured interview [ALS Functional Rating Scale revised form (ALSFRS-R) (23)] for all patients and with functional testing, [Hammersmith Functional Motor Scale Expanded (HFMSE) (24)] for SMA types II and III. Since HFMSE is not an appropriate test for the evaluation of physical function in SMA type I, functional testing for type I was not included in this study. In ALSFRS-R, scores reach from zero (maximum impairment of physical function) to 48 (no impairment of physical function). In HFMSE, scores range from zero (maximum impairment) to 66 (no impairment).

### Statistics

The statistical analysis was performed with SPSS 25.0. To test for normal distribution, Shapiro-Wilks-test was performed and accordingly, parametric tests were used: Friedman's ANOVA for repeated-measures, Mann-Whitney test for two independent groups, Kruskal-Wallis test for more than two independent groups and Kendall's Tau for correlations. A threshold of *p* < 0.05 (two-tailed) was used for statistical interference.

## Results

### Well-Being at Study Entry

At study entry, patients exhibited a significantly higher gQoL than healthy controls (*U* = 92.50, *z* = −2.627, *p* = 0.010, *r* = −0.43). The same was true for the subgroup of pediatric patients (*U* = 26.00, *z* = −2.327, *p* = 0.023, *r* = −0.50). For the subgroup of adult patients, no significant differences compared to controls were found. In addition, no significant differences in qQoL at study entry could be found between SMA types I, II and III or between “walkers,” “sitters” and “non-sitters.”

The level of depressiveness in patients at study entry did not differ significantly from controls (*U* = 153.50, *z* = 0.220, *p* = 0.829, *r* = 0.04). Both reported a median value of 35.0% on a scale from 25.0% (least depressive) to 100% (most depressive) in ADI-12. In accordance to the cut-offs provided for ADI-12, four (19.0%) patients showed at least mild depressive symptoms. Those four patients, two of them with severe depressive symptoms, were all adult, accounting for 28.6% of the adult subgroup. In the adult subgroup of matched controls, 33.3% presented with severe depressiveness. All pediatric patients and controls scored below the cut-off for depressive symptoms. At study entry, no significant differences in depressiveness were found between SMA types I, II and III or between “walkers,” “sitters” and “non-sitters.”

Concerning HRQoL, patients differed from healthy controls in two out of nine dimensions of SF-36 at start of therapy. These were the dimensions of lower “physical function” (*U* = 80.00, *z* = 2.390, *p* = 0.020, *r* = 0.53) and lower “general health” (*U* = 91.00, *z* = 3.156, *p* = 0.001, *r* = 0.71). For the other dimensions, including “mental health,” “social functioning,” and “role-limitations due to physical health” no differences were seen.

### Well-Being Over the Course of 6 Months

**Figure 1:**
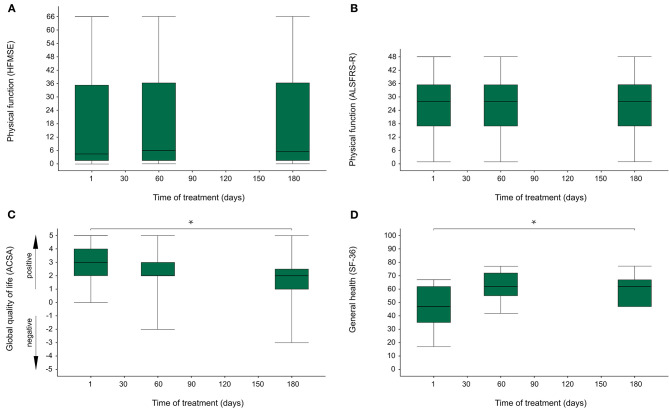
Physical function, global quality of life and rating of general health over the first 6 months of treatment with nusinersen. **(A)** *N* = 21; Physical function (HFMSE) in SMA types II and III was evaluated with Hammersmith Functional Motor Scale Expanded (HFMSE), where a value of 0 indicates minimum physical function, and a value of 66 indicates maximum physical function; physical function did not change significantly over the first 6 months of treatment with nusinersen [χ^2^(2) = 5.042, *p* = 0.080]. **(B)** *N* = 25; Physical function in all subgroups was evaluated with ALS Functional Rating Scale revised form (ALSFRS-R), where a value of 0 indicates minimum physical function, and a value of 48 indicates maximum physical function; physical function did not change significantly over the first 6 months of treatment with nusinersen [χ^2^(2) = 3.000, *p* = 0.223]. **(C)** *N* = 21; Global quality of life (gQoL) in all subgroups was evaluated with Anamnestic Comparative Self-Assessment (ACSA), where values above zero (max. 5) indicate a positive well-being, while values below zero (min. −5) indicate a negative well-being; gQoL decreased significantly over the first 6 months of treatment with nusinersen [χ^2^(2) = 6.30, *p* = 0.043]. **(D)** *N* = 11; Rating of general health in adult SMA patients was assessed with Short Form 36 Health Survey (SF-36), where a value of 0 indicates the worst possible rating of general health, while a value of 100 indicates the best possible rating of general health; the rating of general health increased significantly over the first 6 months of treatment with nusinersen [χ^2^(2) = 7.80, *p* = 0.021]. *indicates a significant change of values over time in Friedman's ANOVA.

In this cohort, there was no significant change of physical function over the first 6 months of treatment [HFMSE χ^2^(2) = 5.042, *p* = 0.080; ALSFRS-R χ^2^(2) = 3.000, *p* = 0.223; Figures 1A,B].

Global QoL significantly decreased longitudinally across all subjects [χ^2^(2) = 6.30, *p* = 0.043] (Figure 1C). Subgroup analyses (adult patients, pediatric patients, SMA types I, II, and III, “walkers,” “sitters” and “non-sitters”), did not yield significant results.

In contrast to the situation at study entry, there was no difference in gQoL between patients and controls after 6 months (*U* = 191.00, *z* = −0.386, *p* = 0.728, *r* = 0.06).

With a stable median value in ADI-12 of 35.0%, depressiveness showed no significant fluctuations over the course of this study [χ^2^(2) = 1.51, *p* = 0.469] across all subjects and also in all subgroups (adult patients, pediatric patients, SMA types I, II, and III, “walkers,” “sitters,” and “non-sitters”).

Concerning HRQoL in adult patients, scores of three out of nine dimensions showed a significant change over 6 months. Firstly, “role-limitations due to physical health” [χ^2^(2) = 6.75, *p* = 0.034], where scores on day 60 were significantly lower than at study entry and after 6 months. Secondly, “general health” [χ^2^(2) = 7.80, *p* = 0.021], where scores on day 60 and after 6 months were significantly higher than at study entry (Figure 1D). Thirdly, “health transition” [χ^2^(2) = 12.18, *p* = 0.002], where scores on day 60 and after 6 months were also significantly higher than at study entry. In the dimensions “social functioning,” “role limitations due to emotional status,” “bodily pain,” “mental health,” “vitality,” and “physical functioning” no change was seen.

### Associations of Well-Being and Clinical Data

**Figure 2:**
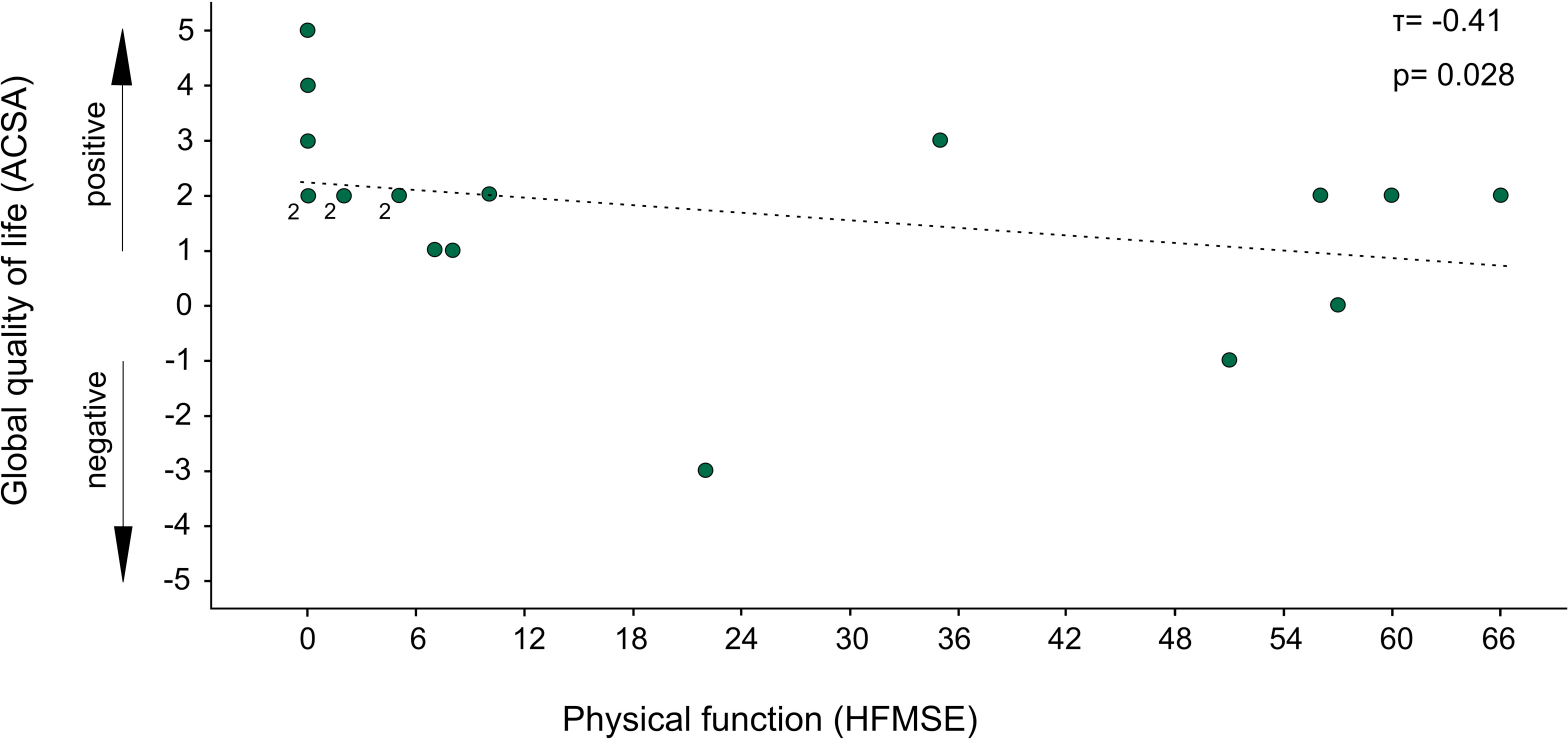
Association of global quality of life with physical function. *N* = 19; Global quality of life (gQoL) was evaluated with Anamnestic Comparative Self-Assessment (ACSA), where values above zero indicate (max. +5) a positive well-being, while values below zero (min. −5) indicate a negative well-being; Physical function was evaluated with Hammersmith Functional Motor Scale Expanded (HFMSE) with values from 0 (minimum physical function) to 66 (maximum physical function); data points labeled “2” represent 2 patients; higher qQoL was significantly associated with lower physical function (τ = −0.41, *p* = 0.028).

Better gQoL correlated with lower physical function (after 6 months; HFMSE: τ = −0.41, *p* = 0.028) across all subjects (Figure 2). Separate analyses of all subgroups found a significant correlation only in the subgroup of non-sitters (after 6 months; HFMSE: τ = −0.67, *p* = 0.021).

**Figure 3:**
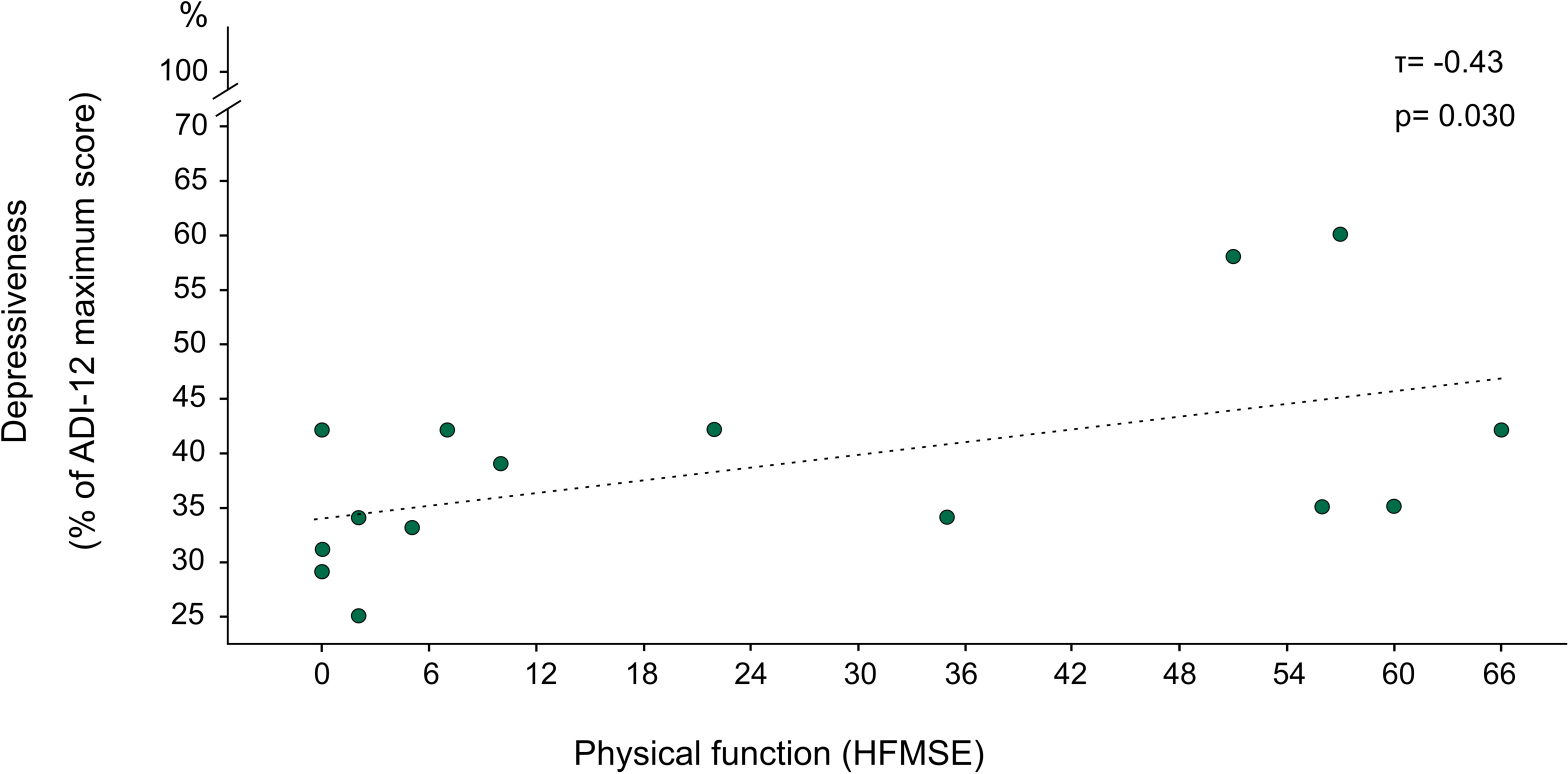
Association of depressiveness with physical function. *N* = 15; Depressiveness was evaluated with ALS Depression Inventory 12 (ADI-12), where the original scale from 12 to 48 was transformed into percentual values of the maximum score, yielding a scale from 25 to 100%. Physical function was evaluated with Hammersmith Functional Motor Scale Expanded (HFMSE) with values from 0 (minimum physical function) to 66 (maximum physical function); lower depressiveness was significantly associated with lower physical function (τ = −0.43, *p* = 0.030).

In accordance, an earlier disease onset was associated with better gQoL (after 6 months; τ = −0.44, *p* = 0.015) across all subjects. In separate analyses of the subgroups (adult patients, pediatric patients, SMA types I, II, and III, “walkers,” “sitters,” and “non-sitters”), no significant correlations were found. Those with a later disease onset exhibited higher levels of depressiveness (after 6 months; τ = −0.49, *p* = 0.018). Furthermore, higher scores for depressiveness were correlated with better physical function (after 6 months; HFMSE: τ = −0.43 *p* = 0.030) (Figure 3). These two correlation analyses did not reveal significant results in separate analyses of the subgroups.

The perception of one's health (dimension “general health” in SF-36) exhibited no correlation with physical function (after 6 months; HFMSE: τ = −0.36, *p* = 0.147).

## Discussion

Despite severe physical restrictions, we revealed a satisfactory gQoL and low levels of depressiveness in this cohort of SMA patients, which is in line with previous observations (6, 7, 9). At study entry, QoL was even higher than in healthy controls and declined during the first half year of therapy with nusinersen. This decline might be tentatively interpreted as a return to a baseline gQoL after a pretherapeutic elevation of gQoL triggered by patients' hopes and expectations toward therapy. This return to a baseline QoL after a few months of transiently elevated QoL would represent a physiological process, which has already been described in association with positive life events (25). Thus, this development does not necessarily imply dissatisfaction or disappointment with therapy. However, a direct negative impact of nusinersen itself on qQoL cannot be excluded entirely, but it is of note that no adverse events were reported. Furthermore, a study by Osmanovic et al. (2) in adult SMA patients under treatment with nusinersen reported high treatment expectations at study entry, which remained stable across 10 months with some negative expectations even decreasing over time. Thus, disappointment or a direct negative effect of nusinersen seem improbable. Nevertheless, it has to be mentioned that measurement of pretherapeutic QoL was not feasible in this study, but should be an important component of future research.

Among patients, better physical function appeared to be a risk factor for worse gQoL and higher levels of depressiveness. This is consistent with previous findings (4) and is explainable by the fact that physical function is worse in those patients with a faster disease progression and consequently a loss of function early on in life. The loss of critical motor abilities at later points in life is thought to make it harder for patients to adapt to a life with SMA, considering that these patients have experienced a life with certain abilities before. Thus, for younger patients, who experienced physical restrictions early on in life, those physical restrictions may have become part of their self-concept with less negative impact on their mental well-being.

Regarding SF-36 values, adult patients differed from controls in only two out of eight dimensions, with lower scores in “physical function” and “general health.” This is in line with a previous study (10), which also reported lower values in the SF-36 dimension “physical function” in adult patients with SMA. However, this study found no significant difference regarding “general health” relative to general population (10). A study in adult patients that focussed only on pain found normal scores in the domain “bodily pain,” which did not differ from the general population (26). This conforms with our findings.

According to our results in the SF-36 domains “general health” and “health transition” (the latter comparing the current health to 1 year ago), adult patients perceived an improvement of health over the course of therapy. This is not in contrast to our finding of declining subjective gQoL, considering that this finding applies for the entire cohort of adult and pediatric patients, while the finding of approved subjective health only applies for adult patients. In this subgroup no significant decline of gQoL could be detected. Furthermore, subjective QoL and HRQoL (including perceived health) are two distinct entities, which can also diverge, as previously shown in patients with ALS (12). As mentioned, there are only very few studies that investigated subjective self-reported quality of life in SMA. This restricts a direct comparison to results of other studies, focusing solely on HRQoL. Still, there is an inevitable need for the inclusion of subjective measures of QoL in clinical studies, since they depict the patients' perception without making further, probably flawed, assumptions on the impact of health on QoL.

It can be discussed whether the perceived improvement in health may also be triggered to some extent by patients' hopes and expectations toward therapy. However, Osmanovic et al. found no influence of treatment expectations on treatment outcome in adult patients treated with SMA (2). Regardless of this, an improvement of subjective experience of health may represent a major impact of nusinersen on SMA patient's life.

### Limitations

Some shortcomings have to be discussed. We investigated a relatively small sample size including pediatric and adult patients with different types of SMA, which may limit the generalizability of our results. Accordingly, subgroup analyses have to be regarded with caution owing to the unbalanced and even smaller group sizes. However, considering SMA as a rare disease, higher sample sizes are hard to acquire, in particular in monocentric approaches. In addition, our follow-up investigations were conducted over a period of 6 months and we cannot exclude significant improvements in motor function along with possible positive effects on QoL after this period of time, in particular in older patients with SMA II and III. Thus, a longer follow-up would be desirable for future studies. To warrant comparability within one study design, we mainly used psychometric measures designed for adult patients with however limited evidence on reliability and validity in a pediatric population. Whereas, the ADI-12 has only been validated for adult patients, a previous study demonstrated good validity of the ACSA in adolescents (eleven to 18 years of age) (27), supporting its use in studies with a great dispersion of age as in our study. Especially in younger patients, proxy estimation by caregivers would have added to our understanding of patients' QoL and affective state, and needs further exploration in future studies. As mentioned, a further limitation in our study is the lack of data on QoL prior to day one of treatment. We therefore cannot exclude alterations in baseline QoL prior to the first application of nusinersen, owing to hopes and expectations toward therapy. Thus, future multicenter studies with homogeneous samples and longer study courses are needed, which also consider QoL measures prior to treatment with nusinersen.

### Conclusion

In conclusion, over the first 6 months of therapy with nusinersen, SMA patients reported a subjective feeling of improved health whereas subjective well-being did not positively change under treatment.

## Data Availability Statement

The raw data supporting the conclusions of this article will be made available by the authors, without undue reservation.

## Ethics Statement

The studies involving human participants were reviewed and approved by Ethics Committee of Ulm University. Written informed consent to participate in this study was provided by the participants' legal guardian/next of kin.

## Author Contributions

LM was responsible for data collection, statistical analysis, data interpretation, and writing the manuscript. BW coordinated the study and critically revised the manuscript. CW was responsible for planning and coordination of the study, as well as critically revising the manuscript. SP collected data and critically revised the manuscript. SW and ZU were involved in coordination of the study. HG critically revised the manuscript. AL was involved in planning of the study and critical revision of the manuscript. DL was responsible for planning of the study, coordination of the study, statistical analysis, and writing the manuscript. All authors contributed to the article and approved the submitted version.

## Conflict of Interest

BW and ZU received honoraria from Biogen for lectures and consultation, respectively. CW received honoraria from Biogen (as an Advisory board member and for lectures) and from Hoffmann-La Roche (as a consultant and Advisory board member). She also received travel expenses from Biogen. AL received honoraria from AB Science, Biogen, Cytokinetics, GSK, Orion Pharma, Novartis, Tau Rx Therapeutics Ltd., TEVA Pharmaceuticals, Mitsubishi, and Hoffmann-La Roche. The remaining authors declare that the research was conducted in the absence of any commercial or financial relationships that could be construed as a potential conflict of interest.
