# Supplementary material for: Quality of Life in SMA Patients Under Treatment With Nusinersen
Source: Front Neurol. 2021 Mar 29;12:626787. doi: 10.3389/fneur.2021.626787 (PMC8039289; doi:10.3389/fneur.2021.626787)
Supplement: Supplementary file 1 [file Table_1.DOCX]

Supplementary Material

# Supplementary Table 1

Median scores in PedsQL at study entry

N (patients) = 8; N (controls) = 8; PedsQL= Paediatric Quality of Life Inventory; u.quart. = upper quartile; l.quart. = lower quartile; interqu. = interquartile range; max.= maximum; min.= minimum; PF = physical functioning; EF =emotional functioning; SF = social functioning; ScF = school functioning; PhyHSS = Physical Health Summary Score; PsyHSS = Psychosocial Health Summary Score; total = total score; *= significant difference between patients and controls; **= highly significant difference between patients and controls; this table reports the median scores in the dimensions of PedsQL (a measure of paediatric HRQoL) of paediatric patients with SMA at start of treatment with nusinersen. Median scores of patients and controls were compared with the Mann-Whitney test and marked with stars (*) in case of a significant difference

| dimension | median | u.quart. | l.quart. | interqu. | max. | min. | median control |
| --- | --- | --- | --- | --- | --- | --- | --- |
| PF | 23.5 | 30.5 | 17.2 | 13.3 | 31.0 | 6.0 | 94.0** |
| EF | 72.5 | 80.0 | 61.3 | 18.8 | 80.0 | 60.0 | 67.0 |
| SF | 77.5 | 88.8 | 66.3 | 22.5 | 92.0 | 40.0 | 90.0 |
| ScF | 75.0 | 83.8 | 66.3 | 17.5 | 85.0 | 60.0 | 82.5 |
| PhyHSS | 23.5 | 30.5 | 17.2 | 13.3 | 31.0 | 6.0 | 94.0** |
| PsyHSS | 76.7 | 79.7 | 67.1 | 12.6 | 63.0 | 57.0 | 77.5 |
| total | 63.0 | 67.0 | 56.2 | 10.8 | 69.0 | 44.0 | 80.4** |

# Supplementary Table 2

Median scores in PedsQL over the first 6 months of treatment with nusinersen

N = 8; PedsQL= Paediatric Quality of Life Inventory; u.quart. = upper quartile; l.quart. = lower quartile; interqu. = interquartile range; PF = physical functioning; EF = emotional functioning; SF = social functioning; ScF = school functioning; PhyHSS = Physical Health Summary Score; PsyHSS = Psychosocial Health Summary Score; total = total score; F-ANOVA = Friedman’s ANOVA; this table reports the median scores of the dimensions of PedsQL (a measure of paediatric HRQoL) in paediatric patients with SMA over the course of treatment nusinersen. The right column analyses the significance of change of scores over time, using F-ANOVA

| dimension | median | | | F-ANOVA |
| --- | --- | --- | --- | --- |
|  | day 1 | day 60 | day 180 |  |
| PF | 23.5 | 18.8 | 22.0 | p = 0.902 |
| EF | 72.5 | 72.5 | 80.0 | p = 0.508 |
| SF | 77.5 | 77.5 | 80.0 | p = 0.519 |
| ScF | 75.0 | 72.5 | 80.0 | p = 0.497 |
| PhyHSS | 23.5 | 18.8 | 22.0 | p = 0.902 |
| PsyHSS | 76.7 | 74.4 | 80.0 | p = 0.206 |
| total | 63.0 | 62.2 | 63.6 | p = 0.417 |
